# Integration of pharmacology, molecular pathology, and population data science to support precision gastrointestinal oncology

**DOI:** 10.1038/s41698-017-0042-x

**Published:** 2017-12-06

**Authors:** Shuji Ogino, Iny Jhun, Douglas A. Mata, Thing Rinda Soong, Tsuyoshi Hamada, Li Liu, Reiko Nishihara, Marios Giannakis, Yin Cao, JoAnn E. Manson, Jonathan A. Nowak, Andrew T. Chan

**Affiliations:** 1 Program in MPE Molecular Pathological Epidemiology, Department of Pathology, Brigham and Women’s Hospital, Harvard Medical School, Boston, MA USA; 2 Department of Epidemiology, Harvard T.H. Chan School of Public Health, Boston, MA USA; 3 Department of Oncologic Pathology, Dana-Farber Cancer Institute, Harvard Medical School, Boston, MA USA; 4 Department of Nutrition, Harvard T.H. Chan School of Public Health, Boston, MA USA; 5 Department of Biostatistics, Harvard T.H. Chan School of Public Health, Boston, MA USA; 6 Broad Institute of MIT and Harvard, Cambridge, MA USA; 7 Department of Medical Oncology, Dana-Farber Cancer Institute, Boston, MA USA; 8 Department of Medicine, Brigham and Women’s Hospital, Harvard Medical School, Boston, MA USA; 9 Clinical and Translational Epidemiology Unit, Massachusetts General Hospital, Boston, MA USA; 10 Division of Gastroenterology, Massachusetts General Hospital, Harvard Medical School, Boston, MA USA; 11 Division of Preventive Medicine, Department of Medicine, Brigham and Women’s Hospital, Harvard Medical School, Boston, MA USA; 12 Channing Division of Network Medicine, Brigham and Women′s Hospital, Harvard Medical School, Boston, MA USA

## Abstract

Precision medicine has a goal of customizing disease prevention and treatment strategies. Under the precision medicine paradigm, each patient has unique pathologic processes resulting from cellular genomic, epigenomic, proteomic, and metabolomic alterations, which are influenced by pharmacological, environmental, microbial, dietary, and lifestyle factors. Hence, to realize the promise of precision medicine, multi-level research methods that can comprehensively analyze many of these variables are needed. In order to address this gap, the integrative field of molecular pathology and population data science (i.e., molecular pathological epidemiology) has been developed to enable such multi-level analyses, especially in gastrointestinal cancer research. Further integration of pharmacology can improve our understanding of drug effects, and inform decision-making of drug use at both the individual and population levels. Such integrative research demonstrated potential benefits of aspirin in colorectal carcinoma with *PIK3CA* mutations, providing the basis for new clinical trials. Evidence also suggests that *HPGD* (15-PDGH) expression levels in normal colon and the germline rs6983267 polymorphism that relates to tumor *CTNNB1* (β-catenin)/*WNT* signaling status may predict the efficacy of aspirin for cancer chemoprevention. As immune checkpoint blockade targeting the *CD274* (PD-L1)/*PDCD1* (PD-1) pathway for microsatellite instability-high (or mismatch repair-deficient) metastatic gastrointestinal or other tumors has become standard of care, potential modifying effects of dietary, lifestyle, microbial, and environmental factors on immunotherapy need to be studied to further optimize treatment strategies. With its broad applicability, our integrative approach can provide insights into the interactive role of medications, exposures, and molecular pathology, and guide the development of precision medicine.

## Introduction–emergence of precision medicine

Science is composed of specific fields of research, which are structured to organize education, training, and scientists themselves. By its nature, science never stops evolving. The medical and health sciences are no exception. As science continuously progresses with the development of concepts, methods, and discoveries, new scientific paradigms and fields emerge, which may augment or replace existing ones.

A good example of this phenomenon is the recent development of precision medicine, which has attracted heated attention, particularly in the field of oncology, with the hope of individualized patient treatment and care. In addition to precision treatment, precision prevention is a part of precision medicine.^1–4^ However, to realize the promise of precision medicine, the development of supporting method-based disciplines is needed. There have been growing concerns about the validity of published study findings^5,6^ and, unless we take action, this problem will only be exacerbated in the era of big data and omics research. Rigorous and standardized research methods are essential for generating reproducible data and generalizable knowledge, to facilitate the realization of precision medicine. Furthermore, a better understanding of research methods can improve not only the quality of research publications but also peer-review processes and evaluation of evidence in the literature. Therefore, we cannot over-emphasize the importance of the development of method-based science.

Pathology is a discipline that concerns pathogenic mechanisms, as well as methods to analyze tissues, cells, and molecules in disease processes. In recent decades, molecular pathology has become dominant, and large amounts of molecular pathology data have accumulated worldwide.^7,8^ The advancement of molecular pathology is not only transforming disease classification schemes, but is also generating opportunities for improved prevention and treatment.^7,8^ However, to optimally utilize molecular pathologic data, there is a pressing need to integrate data science into pathology.

**Fig. 1:**
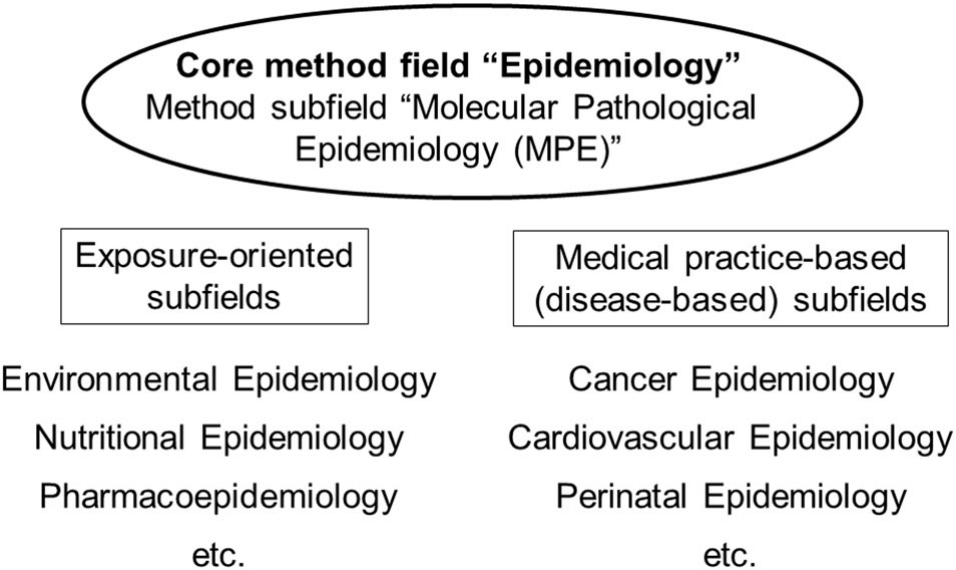
Structure of epidemiology and its subfields. Since the formation of the field of epidemiology, a number of subfields have emerged to specialize into particular subject matters, including detailed analyses of exposure factors (depicted on the left) and detailed analyses of health outcomes or diseases (depicted on the right). Six such subfields among many are shown. In addition, a method subfield "molecular pathological epidemiology (MPE)" has been developed under the core method field of epidemiology. MPE can be applied to any exposure and disease settings, and can be integrated with any other subfield of epidemiology

Epidemiology is a method-based discipline that concerns not only examining determinants of disease and health outcomes but also the development of data analysis methods. Epidemiologic research provides data to inform evidence-based clinical practice and health policy-making. Essentially every medical study explicitly or implicitly uses epidemiologic principles to produce generalizable scientific knowledge. These facts attest to the importance of epidemiology as a core method-based discipline. Epidemiology has been applied in not only various disease-based fields (to generate subfields such as cancer epidemiology) but also research areas that focus on specific endogenous or exogenous health-related factors (to generate subfields such as pharmacoepidemiology) (Fig. 1). As various subfields emerge and evolve, the field of epidemiology constantly transforms to generate new concepts and address ever-changing practical and educational needs.

In this article, we discuss the value and implications of integrating pharmacology, molecular pathology, and epidemiology, utilizing gastrointestinal cancers as a disease model. Our intent is to illustrate the clinical and public health implications of this integrative concept that can be instrumental in advancing the field of cancer precision medicine.

## Molecular pathological epidemiology

**Fig. 2:**
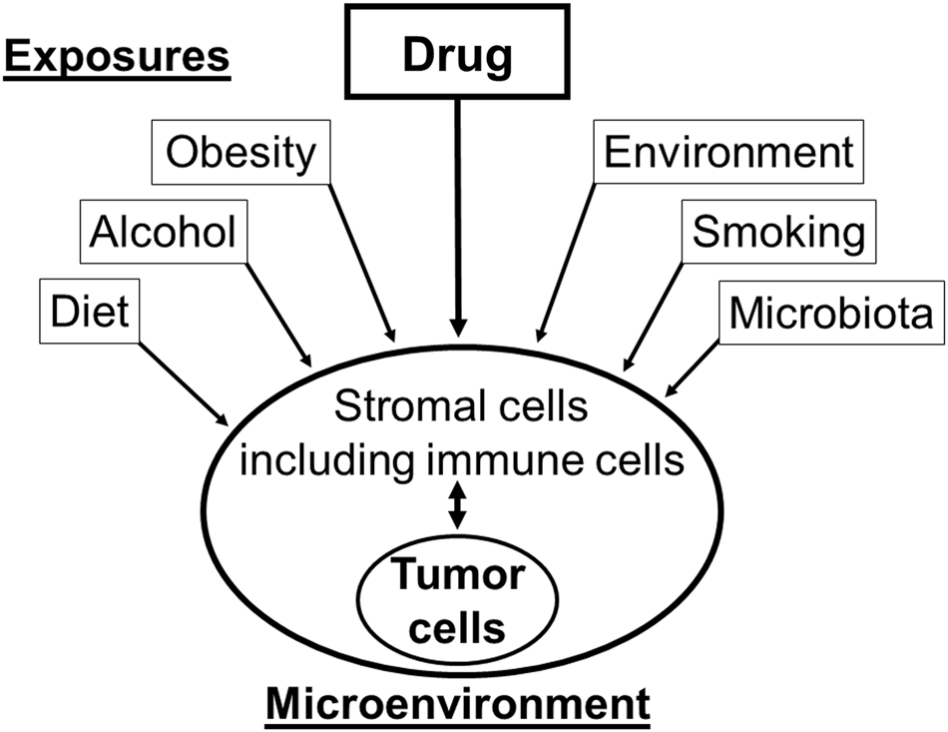
Influences of various exposures on pathogenic process. A wide variety of endogenous and exogenous factors (including drugs), individually or in combination, can modify phenotypes of cancer, leading to interpersonal heterogeneity. The molecular pathological epidemiology (MPE) approach utilizes integrated analyses of these exposures and tumor phenotypes to improve our understanding of tumor development and progression. Of note, for the sake of simplicity, this illustration does not depict complex interactions between the exposure factors

Although molecular pathology was incorporated into the broad umbrella of molecular epidemiology in the 1990s, a substantial gap between molecular pathology and epidemiology resulted in limited interest in developing integrated approaches.^9^ To address this, a single unified field of molecular pathological epidemiology (MPE), which integrates research approaches in molecular pathology and epidemiology, has emerged.^10–12^ The basic principle of MPE resides in the fact that many endogenous and exogenous factors (commonly referred to as "exposures"), in combination, modify phenotypes of diseases including cancer (Fig. 2); hence, better understanding of cancer requires comprehensive analyses of exposures and tumor phenotypes. In MPE research, an exposure or risk factor can be connected to specific pathogenic signatures.^12^ As a more homogeneous group of patients (i.e., disease subtype based on common pathogenic features) have similar etiologies, the MPE approach can yield a more accurate risk measure.^11,12^ This new research framework of MPE drives the development of epidemiological and statistical methods.^13–18^ Although challenges exist in MPE,^11^ the paradigm of MPE has been widely accepted,^19–29^ and discussed at various international meetings.^30–33^ MPE can also provide evidence linking drugs and disease outcomes,^34–37^ thereby supporting the precision medicine initiative. As a versatile method-based discipline, MPE can be applied to any disease setting, and further integrated with other investigative fields. To date, the MPE approach has been most commonly used in research of cancers, especially colorectal cancer.^11,38^

## Pharmacoepidemiology

**Fig. 3:**
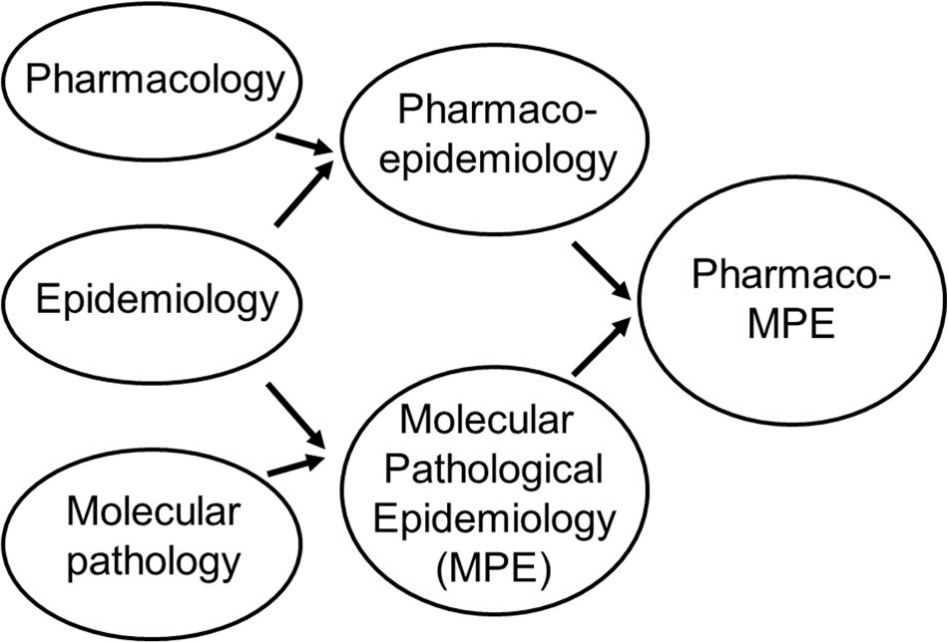
Trans-multidisciplinary integration of pharmacology, epidemiology, and molecular pathology. The integration of pharmacology and epidemiology has generated pharmacoepidemiology, while the integration of molecular pathology and epidemiology has generated molecular pathological epidemiology (MPE). We propose the integration of pharmacoepidemiology and MPE to generate pharmaco-MPE

Pharmacoepidemiology is the study of the use and effects of drugs and other medical devices in populations.^39^ In the 1980s, the field of pharmacoepidemiology arose as the integration of pharmacology and epidemiology (Fig. 3), primarily to improve drug surveillance programs for ensuring safe and effective medication use.^39^ Hence, drug surveillance and effectiveness research has been a major focus of pharmacoepidemiology. With the precision medicine initiative,^40^ molecular diagnostics has become an important part of clinical practice and decision making, especially to guide medication use. Therefore, there are ample opportunities for pharmacoepidemiology to expand and incorporate advances of molecular pathology.

## Value of integrating pharmacoepidemiology and MPE

**Fig. 4:**
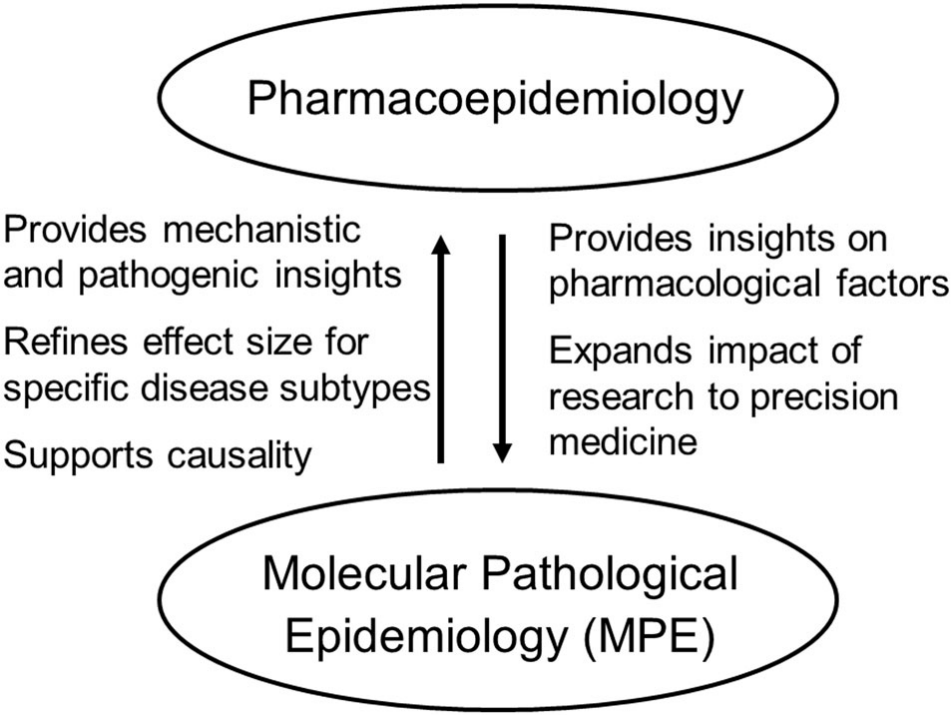
Collaborative relationship between pharmacoepidemiology and molecular pathological epidemiology (MPE). Both are subfields of epidemiology, and cover the entire spectrum of human diseases. Both fields can be synergized to create an integrative field of pharmaco-MPE, which can further enhance research and education for precision medicine

When we study pharmacologic factors and molecular pathology using the epidemiologic principles, the integration of pharmacoepidemiology and MPE (herein referred to as pharmaco-MPE) can be considered (Fig. 3). What is the value of this integration? Taking advantage of the complementary, synergistic strengths of pharmacoepidemiology and MPE (as illustrated in Fig. 4), the integrative approach can provide new insights into interactions of drug, environmental, and host factors in pathologic processes, thereby helping personalize therapies and advance precision medicine.

For the past decades, research on inter-individual differences in effects of drugs has become increasingly relevant. In precision medicine, it is critical to understand the effects of drugs in relation to the spectrum of molecular pathological characteristics.^41^ In fact, the integrative MPE approach has been useful in investigating effects of exposures, including drugs, and their interactive effects with disease molecular pathology (as explained in the next section).^38^

With the precise definition of epidemiology, any clinical investigation (regardless of its design) explicitly or implicitly uses epidemiologic principles with a goal of obtaining generalizable knowledge, and therefore can be regarded as epidemiologic research. Despite this fact, many investigators do not have adequate knowledge of epidemiologic principles, which has led to the situation that "most published research findings are false".^5^ Thus, epidemiologic principles that can enhance scientific rigor are of paramount importance. Essentially all clinical studies on drugs (including clinical trials) utilize epidemiologic principles, and can be regarded as pharmacoepidemiologic research. By these definitions, clinical studies that examine both drugs and molecular pathology can be regarded as pharmaco-MPE research. With this "pharmaco-MPE" designation, any investigators who examine drugs, molecular pathology, and clinical outcomes should be aware that they must utilize appropriate multidisciplinary expertise, and follow research standards of all of the component fields, i.e., pharmacology, molecular pathology, and epidemiology. While invalid results from poorly-designed studies confuse both researchers and practitioners, evidence from high quality pharmaco-MPE research can potentially improve clinical practice and guide future research.

## Integrative research model on aspirin and colorectal cancer

In this section, we discuss studies on aspirin and colorectal cancer as a pharmaco-MPE research model, to illustrate its potential for impacting clinical practice. Colorectal cancer remains a major cause of morbidity and mortality. Colorectal carcinoma represents a group of molecularly heterogeneous tumors with varying combinations of somatic genetic and epigenetic changes,^42–47^ as well as varying degrees of immune response to tumor.^48–53^ As the colorectum is a long organ with differing contents and microbiota along its length, tumor characteristics vary according to its subsites.^54–58^

Accumulating evidence supports the anti-cancer effect of aspirin, one of the non-steroidal anti-inflammatory drugs (NSAIDs).^59–61^ Aspirin is a modulator of immunity and inflammation.^49,62,63^ A recent study has shown a stronger cancer-preventive effect of aspirin for colorectal cancer compared to other major cancer types.^64^ In addition, aspirin may be used for colorectal cancer therapy.^65,66^ As it is conceivable that the effect of aspirin differs by tumor subtype, the pharmaco-MPE approach could be useful to determine which individuals would gain more benefits from aspirin therapy. Previous pharmaco-MPE studies have utilized population-based datasets to examine potential influences of common medications (including aspirin) on incidence and progression of colorectal cancer subtypes as classified by tissue biomarkers including *PTGS2* (cyclooxygenase 2) expression.^34–37,67–72^ Notably, a pharmaco-MPE study provided evidence for a strong beneficial effect of aspirin for *PIK3CA*-mutated colorectal cancer but not for *PIK3CA*-wild-type cancer.^71^ Hence, *PIK3CA* mutation in colorectal cancer may be a predictive biomarker for response to aspirin, while its prognostic role may not be significant.^73–79^ The findings have been subsequently tested in independent datasets,^80–83^ systematic review and meta-analyses,^73,84^ and in vitro experiments on colon and breast cancer cells.^85,86^ Although most of these follow-up studies support the original observations in the human population, new prospective trials on aspirin are needed to validate the findings in addition to the existing trials on aspirin.^63,65,87^ Pharmaco-MPE studies on the association of postdiagnosis aspirin use with colorectal cancer survival according to other tumor molecular markers are also warranted. For example, alterations in the *TGFB1* (transforming growth factor beta 1, TGF-beta) signaling have been implicated in colorectal carcinogenesis,^88^ and loss-of-function mutations in *TGFBR2* (*TGFB1* receptor 2) may serve as prognostic biomarkers for colorectal cancer patients. Interestingly, this signaling pathway interacts with the PI3K-*AKT*-*MTOR* pathway,^88^ which may modify the anti-tumor effects of aspirin, as described.

With regard to primary prevention, MPE research may help identify a biomarker to predict efficacy of aspirin. Studies provide evidence to support varying cancer-preventive effect of aspirin for different tumor subtypes.^34–36,67^ Notably, aspirin has been associated with lower risk for colorectal cancer with higher expression of *HPGD* (hydroxyprostaglandin dehydrogenase 15-(NAD), 15-PGDH) in normal adjacent colon mucosa.^72^ *HPGD*, a prostaglandin-degrading enzyme, has been shown to down-regulate the proinflammatory reaction.^89,90^ *HPGD* in normal colon in cancer-free individuals may be a candidate biomarker to predict the efficacy of aspirin for primary cancer prevention.^72^ In addition, in vivo imaging technologies (such as low-coherence enhanced backscattering spectroscopy^91^) can be utilized to evaluate cellular molecular changes associated with use of aspirin or other drugs.^92^ Another candidate predictor of aspirin efficacy is the rs6983267 single nucleotide polymorphism in 8q24. A study has shown an association of aspirin with lower colorectal cancer risk only in individuals carrying the minor T allele (rs6983267), and a specificity of this association for the *CTNNB1* (beta-catenin)-positive *WNT* signaling activated tumor subtype.^67^ Together with the gene-environment interaction analysis,^93^ integrated analyses of drugs, germline genetics, and somatic molecular alterations will further shed lights on inter-individual differences in drug effects.

As exemplified by pharmaco-MPE research on aspirin, integration of existing data on medications, molecular pathology, and clinical outcomes can help discover a new biomarker-drug combination to predict drug efficacy in specific populations stratified by the biomarker. Pharmaco-MPE can be applied for other medications including statins, metformins, and bisphosphonates. For example, statins, 3-hydroxy-3-methylglutaryl-coenzyme A (HMG-CoA) reductase inhibitors, can suppress the RAS protein and the Rho family of proteins,^94^ which are strongly implicated in carcinogenesis. Therefore, pharmaco-MPE studies on the association of statin use with colorectal cancer incidence and survival according to *KRAS* mutation status have been reasonably conducted.^69,95^ Because molecular pathology tests have become part of routine clinical practice to guide decision making, molecular pathology data will rapidly accumulate among various populations, and can be utilized in pharmaco-MPE research.^41^ Findings from pharmaco-MPE research can guide not only drug repurposing^96^ for efficient utilization of common medications, but also tailored drug abstention or dosing adjustment to avoid potential side effects that can be predicted from individual’s characteristics.

## Study designs

**Fig. 5:**
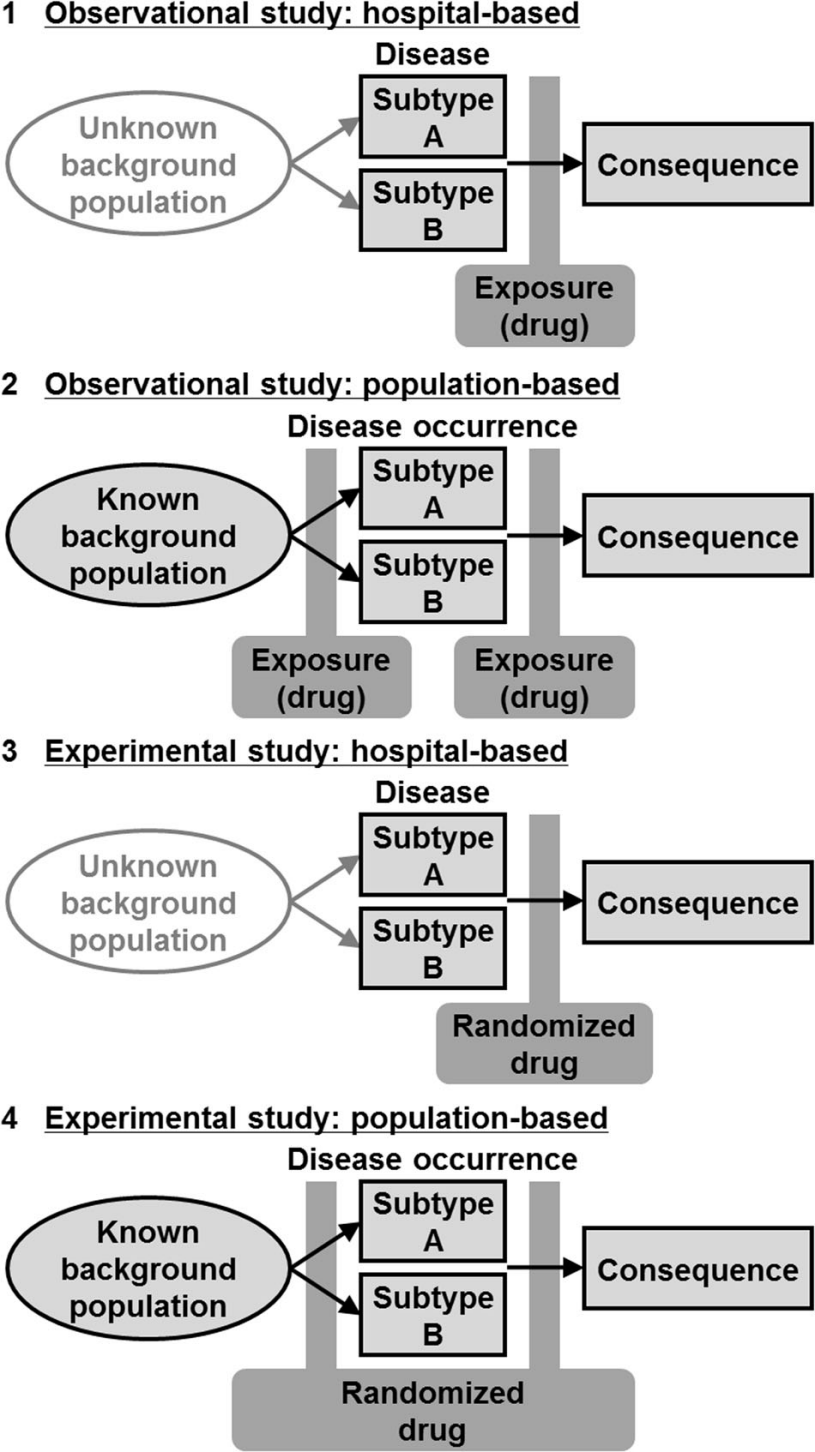
Study Designs in pharmaco-molecular pathological epidemiology (MPE) research. Arrows indicate time sequence. Here, the disease of interest is sub-classified based on pathogenic signatures into binary subtypes A and B for simplicity. Note that multiple subtypes can be evaluated in pharmaco-MPE research. Analyses can be conducted to assess effects of a drug of interest on the occurrence and/or consequential event (such as death) of a specific disease subtype. In the MPE research framework, a difference in the associations between disease subtypes is assessed. Panels indicate specific designs (with the corresponding column number in Table 1) as follows: 1, observational hospital-based design; 2, observational population-based design; 3, experimental hospital-based trial; and 4, experimental population-based trial

Better understanding of study designs, including their strengths and weaknesses, is a prerequisite for rigor and reproducibility in science. There are four main study designs in pharmaco-MPE research (Fig. 5). Characteristics of the four designs are compared in Table 1. An observational study on a subject sample drawn from one or more institutions is herein referred to as the "observational hospital-based design" (Fig. 5a). Compared to the three other designs, internal, and external validities (i.e., generalizability) of this study design's findings may be relatively limited.

**Table 1:** Comparisons of study designs in pharmaco-molecular pathological epidemiology (MPE)

Study design (panel number in Fig. 5)	Observational study: hospital-based^a^ (1)	Observational study: population-based^a^ (2)	Experimental trial study: hospital-based^b^ (3)	Experimental trial study: population-based^b^ (4)
Typical study base population	Patients with a certain disease in question from a single to several hospitals (or institutions)	General population (broadly selected population)	Patients with a certain disease in question from a single to several hospitals (or institutions)	General population. Study population may be a highly selected group of individuals, to conduct a trial study
Typical study outcome	Consequence of the disease	Disease occurrence or consequence in a prospective cohort design. Disease prevalence in a case-control design. A prospective design enables assessment of occurrences of a number of diseases	Consequence of the disease	Disease occurrence or consequence. This design enables assessment of occurrences of a number of diseases
Use of a certain hospital or healthcare system in selection of study subjects	Yes	Usually no (but can be yes)	Yes	Usually yes, to conduct a trial study
Issues to consider for potential sources of sample selection bias	• The source population which has given rise to cases is unaccounted and inexplicable	• The source population which will give (or has given) rise to cases can be characterized	• The source population which has given rise to cases is unaccounted and inexplicable	• The source population which will give (or has given) rise to cases can be characterized
	• Selection bias may arise based on study inclusion criteria, healthcare coverage, geographic restriction, initial recruitment rate, and/or stability of follow-up	• Selection bias may arise based on study inclusion criteria, initial recruitment rate, and/ or stability of follow-up	• Selection bias may arise based on study inclusion criteria, incentives to enroll (or not to enroll), healthcare coverage, geographic restriction, initial recruitment rate, and/or stability of follow-up	• Selection bias may arise based on study inclusion criteria, incentives to enroll (or not to enroll), initial recruitment rate, and/or stability of follow-up
Sample size	Usually small; can be large	Usually large	Usually small; can be large	Usually large
Molecular pathologic analyses of cases	Maybe not difficult in a study with one to a few hospitals that have protocols in place for collection of molecular pathology data	Usually difficult because cases are typically seen in many different hospitals	Maybe not difficult in a study with one to a few hospitals that have protocols in place for collection of molecular pathology data	Usually difficult because cases are typically seen in many different hospitals
Medication data	Available through hospital records and/or recall of participants	Available through hospital records, and/or recall of participants.	Randomized medication data are always available. Other medication data are usually available through hospital records and/or recall of participants.	Randomized medication data are always available. Other medication data are usually available through hospital records and/or recall of participants.
Typical cost to establish a base study cohort and perform a pharmaco-MPE study	Least expensive	Very expensive (less expensive in a case-control design than a prospective cohort design)	Expensive	Most expensive
Issues that may affect internal and external validities	• Internal validity may be limited by residual and unmeasured confounding	• Internal validity may be limited by residual and unmeasured confounding, and/or recall bias	• External validity may be limited by subject selection bias, and/or small sample size	• External validity may be limited by subject selection bias
	• External validity may be limited by subject selection bias, and/ or small sample size	• External validity may be limited by subject selection bias.		• Typically, higher generalizability compared to the other designs
	• Typically, lower generalizability compared to the other designs			

^a^ The distinction between these two observational designs can be ambiguous

^b^ The distinction between these two experimental trial designs can be ambiguous

A second study design, herein referred to as "observational population-based design" (Fig. 5b), is intended to analyze a sample of individuals based on large populations (but not on a small number of institutions), so that findings will be likely generalizable. The sample sizes of these prospective cohort studies are considerably large, to have a sufficient number of occurrences of the disease of interest. As an alternative to the prospective design, investigators can design a case-control study where they intend to sample representative cases and controls from the same background population.

A third design, an experimental trial design using patients in one or more institutions (Fig. 5c), has the main purpose of removing confounding in the assessment of drug effects with randomized assignment to the drug. Occasionally, dozens of institutions can participate and provide cases to increase the sample size; examples are clinical trials of cooperative groups such as the Alliance in Clinical Trials in Oncology, Pan-European Trials in Alimentary Tract Cancers (PETACC), and the National Surgical Adjuvant Breast and Bowel Project (NSABP). Because of the challenge in securing a large sample in a trial setting, most ongoing trials have sample sizes sufficient to draw reasonable conclusions for the main aim of determining drug effects in the entire trial sample. As molecular subtyping classifies patients into smaller subgroups, statistical inference may be an important challenge in pharmaco-MPE research.

Rarely, an experimental trial is performed on a large sample of individuals who have not had the disease condition in question (Fig. 5d). Its main purpose is to analyze a large subject sample that is not based on a small number of institutions, at the same time removing confounding in the assessment of drug effects. Hence, the generalizability of findings is considered higher than the other designs. A substantial challenge is its cost and labor-intensiveness.

## Challenges and opportunities in pharmaco-MPE

As a subfield of epidemiology, pharmaco-MPE shares many strengths and pitfalls with epidemiology. In addition, the current field of MPE has its own pitfalls, as described above. Major challenges include the paucity of transdisciplinary experts and education programs. One purpose of this article is to promote awareness of new opportunities in this emerging field of pharmaco-MPE, as more investigators with appropriate cross-disciplinary training are needed.

**Fig. 6:**
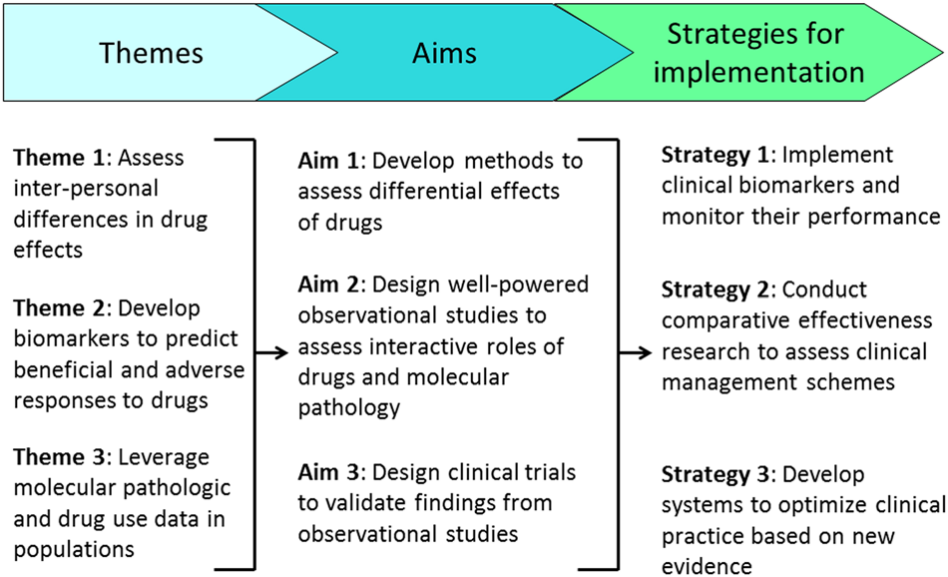
Roadmap for implementing molecular pathologic biomarkers for precision medicine. Three themes are set to launch integrated pharmaco-molecular pathological epidemiology (MPE) research and achieve three specific aims. Based on data obtained by research for the specific aims, Strategies 1 through 3 will help implement, monitor, and optimize tumor biomarker testing for clinical impact

New opportunities in MPE are emerging in the context of a synergistic integration of pharmacoepidemiology and MPE methods, as each subfield has its own unique strengths (Fig. 4). The field of MPE can provide molecular pathologic insights into drug surveillance and safety research where traditional pharmacoepidemiology has a considerable strength. MPE methods also enable investigators to evaluate the effect of a drug in specific disease subtypes, which can improve causal inference. On the other hand, pharmacoepidemiology, which has been developed in close relationships with industries and regulatory agencies (e.g., the U.S. Food and Drug Administration, FDA), can provide not only methods to examine utilization of drugs in populations, but also provide opportunities to extend findings to improve public health policies. Implementation science^97^ has been one of major focuses of pharmacoepidemiology. Hence, the strengths of pharmacoepidemiology and those of MPE are not only complementary but also synergistic in the pharmaco-MPE approach. Once a population-based pharmaco-MPE database is established, a multi-level approach can be possible to combine with germline genetics and other blood biomarkers. Figure 6 illustrates a roadmap of integrative pharmaco-MPE research to conduct rigorous studies, realize precision medicine, and exert clinical impact.

In addition, together with diet and nutrients,^98^ drugs can be used to modulate the microbiota (and the relationship of host immunity to the microbiota), while the microbiota can also modulate drug effects.^99^ Regular use of antibiotics has been associated with a higher risk of colorectal adenoma likely due to pertubation of the gut microbiota.^100^ Investigations into the complex inter-relationship between drugs, the microbiota, immunity, and disease outcomes are important, because changes in the microbiota and immunity can cause not only benefits but also harms to individuals.

Immunotherapeutic strategies have been revolutionizing oncology practice. Recent evidence indicates that mismatch repair (MMR) deficiency, manifested as the microsatellite instability (MSI)-high phenotype, can predict response to immune checkpoint blockade targeting the *CD274* (PD-L1)/*PDCD1* (PD-1) pathway in a wide variety of cancer types including metastatic gastrointestinal cancers.^101^ Hence, the FDA has approved use of the anti-*PDCD1* (PD-1) antibody pembrolizumab for the treatment of mismatch repair-deficient or MSI-high tumors irrespective of primary organ site (https://www.fda.gov/drugs/informationondrugs/approveddrugs/ucm560040.htm; last visited on August 3, 2017). This is the first FDA approval of a drug use based on MMR or MSI test results, attesting to the importance of tumor molecular characteristics in determining response to therapy. There has been ample evidence indicating the important influence of diet and the environment on tumor-immune interactions.^98^ To further optimize treatment strategies and improve clinical outcomes, potential modifying effects of diet, lifestyle, microbial, and environmental factors on immunotherapy should be studied, as new data could inform patient management.

## Conclusion

Given the importance of drugs not only for patient care and public health but also for pharmaceutical industries and the economy at large, the fields of pharmacoepidemiology and pharmaco-MPE can be expected to grow. The process of integrating pharmaco-MPE has been somewhat slow due to disciplinary barriers and the complex nature of the molecular pathology of disease. Nonetheless, research efforts integrating pharmacoepidemiology and MPE are underway. To further advance the field, additional efforts are needed to establish integrated education and training systems. Funding mechanisms must be adapted to ensure fair peer-review for trans- and inter-disciplinary science. Although transdisciplinary research has the potential to make a stronger impact,^102^ its funding success has been lower than traditional scientific approaches.^103^ We anticipate that the value of the integrative science of pharmaco-MPE will become increasingly recognized in the age of precision medicine, as improved understanding of drugs within the context of molecular pathology, and vice versa, can inform the development of customized preventive and therapeutic strategies.

## Use of standardized official symbols

We use HUGO (Human Genome Organisation)-approved official symbols and root symbols for genes, gene products, and gene family, including AKT, CD274, CTNNB1, CYP2D6, HPGD, KRAS, MTOR, PDCD1, PIK3CA, PTGS2, TGFB1, TGFBR2, and WNT; all of which are described in detail at www.genenames.org. Symbols of genes and gene products are italicized, to differentiate from colloquial names that are used only in parenthesis with non-italicized forms. This format enables readers to familiarize the official gene symbols together with common colloquial names.
